# Performance of an Automated Algorithm Grading Surgery-Related Adverse Events According to the Clavien-Dindo Classification: A Systematic Review

**DOI:** 10.7759/cureus.100960

**Published:** 2026-01-06

**Authors:** Mohammed Abutalib Elmobark Gafar, Shashwat Shetty, Muhammad Qaiser Aziz, Eithar Diaeldin Awad Abdelrahman, Mohammed Muatasim Abbas Eltoom, Bilal Ahmad

**Affiliations:** 1 General Surgery, University of EI imam EI Mahdi, Kosti, SDN; 2 Orthopaedics, Hillingdon Hospital, Uxbridge, GBR; 3 Cardiac Surgery, Liaquat National Hospital and Medical College, Karachi, PAK; 4 Pediatric Surgery, Ahfad University for Women, Omdurman, SDN; 5 Anatomy, Omdurman Islamic University, Omdurman, SDN; 6 General Surgery, Dow University of Health Sciences, Civil Hospital Karachi, Karachi, PAK

**Keywords:** automated grading, clavien–dindo classification, machine learning, postoperative complications, surgical outcomes

## Abstract

Postoperative adverse events (AEs) significantly impact patient outcomes and healthcare resources. The Clavien-Dindo Classification (CDC) is widely used to grade surgical complications, but manual grading is labor-intensive and subject to inter-observer variability. Automated algorithms, including rule-based, machine learning (ML), and large language model (LLM)-based natural language processing (NLP) tools, offer scalable solutions for consistent complication grading. A systematic review was conducted following PRISMA 2020 guidelines. Databases searched included PubMed, Embase, Scopus, and Cochrane Library. Studies reporting automated grading of surgery-related AEs using the CDC as a reference, with human validation, were included. Data extraction covered algorithm type, sample size, surgical population, comparator, data source, performance metrics, and outcomes. Three studies met the inclusion criteria, encompassing a total of 1,661 surgical cases. Automated algorithms for Clavien-Dindo Classification (CDC) grading including rule-based systems, machine-learning (ML) models, and large language model (LLM)/natural language processing (NLP) approaches demonstrate high agreement with expert reviewers, with rule-based algorithms achieving Cohen’s κ up to 0.89, ML prediction models reporting discrimination up to an AUC of 0.863 for severe (CDC ≥ III) complications, and LLM/NLP approaches reaching accuracy of approximately 97% and Cohen’s κ up to 0.92. Together, these methods show potential for scalable and, in some settings, near-real-time postoperative complication monitoring. These tools may support clinical decision-making, research, and quality improvement with promising but preliminary applicability across surgical domains. However, conclusions are limited by the small number of available studies and heterogeneity in surgical settings.

## Introduction and background

Postoperative adverse events (AEs) remain a critical challenge in modern surgical practice, significantly contributing to patient morbidity, prolonged hospital stays, readmissions, and overall health care costs. Accurate identification and classification of these complications are essential for quality improvement, benchmarking, and patient safety initiatives. The Clavien-Dindo Classification (CDC) is the most widely used system for grading postoperative complications, stratifying AEs based on the type of therapeutic intervention required, ranging from minor pharmacologic interventions to major surgical procedures or life-threatening events [1]. Despite its widespread adoption, CDC grading is commonly performed via manual chart review, which is labor-intensive, time-consuming, and prone to subjectivity. Inter-observer variability has been documented even among experienced clinicians, potentially compromising the reliability of complication reporting [2].

The advent of electronic health records (EHRs) and computational analytics has created opportunities to automate AE detection and grading. In particular, machine learning (ML) algorithms, natural language processing (NLP) tools, and large language models (LLMs) have demonstrated the ability to extract and classify clinical events from structured and unstructured data sources. However, LLMs also raise concerns regarding hallucinations, limited explainability, dependence on training data quality, and reproducibility, which may affect clinical reliability. Automated approaches offer several advantages, including scalability, consistent application of grading rules, and near-real-time monitoring, potentially reducing reliance on labor-intensive manual review. Recent studies indicate that NLP-based tools can achieve high sensitivity and specificity in identifying postoperative complications from narrative clinical notes [3].

Recent developments in LLMs have further expanded the possibilities for automated CDC grading. These models can process free-text discharge summaries and clinical notes, applying clinical reasoning to assign complication grades with a high degree of concordance with expert reviewers. In one study, LLM-based grading achieved a Cohen’s κ of 0.92 compared with expert human raters, suggesting that AI-driven approaches may approach near-expert performance [4]. However, despite these promising results, challenges remain. Differences in data type (structured vs. unstructured), algorithmic design (rule-based, ML, NLP/LLM), and reference standards contribute to variability in performance metrics. Moreover, most studies are limited to single institutions or surgical specialties, restricting the generalizability of findings. Recent developments in LLMs have further expanded the possibilities for automated CDC grading. While these tools show near-expert agreement with human reviewers, their safe and reproducible deployment requires careful governance. Key considerations include reporting of model version and prompts, temperature and decoding settings, audit trails, data de-identification procedures, human-in-the-loop oversight, calibration against reference standards, and external validation. Incorporating these strategies is essential to ensure methodological rigor, transparency, and alignment with clinical deployment requirements. Although multiple computational approaches have been proposed, there is no consolidated synthesis comparing how different automated methods perform in grading established postoperative complications using the Clavien-Dindo framework. The heterogeneity of algorithms, data sources, and validation strategies remains poorly characterized.

The primary aim of this review was to identify and critically appraise published algorithms for automated post-event Clavien-Dindo grading of postoperative complications. Predictive-only studies, focusing solely on forecasting future complications, were excluded to maintain conceptual consistency. Secondary aims were to describe algorithmic approaches, data sources, validation methods, and reported performance metrics.

## Review

Materials and methods

Search Strategy

This systematic review was conducted in accordance with the PRISMA 2020 guidelines and a completed PRISMA 2020 checklist in Table 1, detailing compliance to ensure methodological transparency and reproducibility [5]. A comprehensive search strategy was implemented across multiple databases, including PubMed, Embase, Scopus, and the Cochrane Library. Search terms combined concepts related to “Clavien-Dindo classification,” “postoperative complications,” “algorithm,” “machine learning,” and “artificial intelligence.” No date restrictions were applied. The final search was conducted on 15 March 2025. Given the rapid evolution of AI/LLM research, no update search was performed prior to submission; this end date was selected to balance feasibility and currency of included studies. Titles and abstracts were screened independently by two reviewers, followed by full-text assessment of potentially relevant articles. Discrepancies were resolved through discussion or consultation with a third reviewer. Data extracted included study design, population, intervention (algorithm type), comparator, reference standard, data source, performance metrics, and key outcomes.

**Table 1:** PRISMA 2020 Checklist From: Page MJ, McKenzie JE, Bossuyt PM, Boutron I, Hoffmann TC, Mulrow CD, et al. The PRISMA 2020 statement: an updated guideline for reporting systematic reviews. BMJ 2021;372:n71. doi: 10.1136/bmj.n71. [5] This work is licensed under CC BY 4.0. To view a copy of this license, visit https://creativecommons.org/licenses/by/4.0/

Section and Topic	Item #	Checklist item
TITLE		
Title	1	Identify the report as a systematic review.
ABSTRACT		
Abstract	2	See the PRISMA 2020 for Abstracts checklist.
INTRODUCTION		
Rationale	3	Describe the rationale for the review in the context of existing knowledge.
Objectives	4	Provide an explicit statement of the objective(s) or question(s) the review addresses.
METHODS		
Eligibility criteria	5	Specify the inclusion and exclusion criteria for the review and how studies were grouped for the syntheses.
Information sources	6	Specify all databases, registers, websites, organisations, reference lists and other sources searched or consulted to identify studies. Specify the date when each source was last searched or consulted.
Search strategy	7	Present the full search strategies for all databases, registers and websites, including any filters and limits used.
Selection process	8	Specify the methods used to decide whether a study met the inclusion criteria of the review, including how many reviewers screened each record and each report retrieved, whether they worked independently, and if applicable, details of automation tools used in the process.
Data collection process	9	Specify the methods used to collect data from reports, including how many reviewers collected data from each report, whether they worked independently, any processes for obtaining or confirming data from study investigators, and if applicable, details of automation tools used in the process.
Data items	10a	List and define all outcomes for which data were sought. Specify whether all results that were compatible with each outcome domain in each study were sought (e.g. for all measures, time points, analyses), and if not, the methods used to decide which results to collect.
	10b	List and define all other variables for which data were sought (e.g. participant and intervention characteristics, funding sources). Describe any assumptions made about any missing or unclear information.
Study risk of bias assessment	11	Specify the methods used to assess risk of bias in the included studies, including details of the tool(s) used, how many reviewers assessed each study and whether they worked independently, and if applicable, details of automation tools used in the process.
Effect measures	12	Specify for each outcome the effect measure(s) (e.g. risk ratio, mean difference) used in the synthesis or presentation of results.
Synthesis methods	13a	Describe the processes used to decide which studies were eligible for each synthesis (e.g. tabulating the study intervention characteristics and comparing against the planned groups for each synthesis (item #5)).
	13b	Describe any methods required to prepare the data for presentation or synthesis, such as handling of missing summary statistics, or data conversions.
	13c	Describe any methods used to tabulate or visually display results of individual studies and syntheses.
	13d	Describe any methods used to synthesize results and provide a rationale for the choice(s). If meta-analysis was performed, describe the model(s), method(s) to identify the presence and extent of statistical heterogeneity, and software package(s) used.
	13e	Describe any methods used to explore possible causes of heterogeneity among study results (e.g. subgroup analysis, meta-regression).
	13f	Describe any sensitivity analyses conducted to assess robustness of the synthesized results.
Reporting bias assessment	14	Describe any methods used to assess risk of bias due to missing results in a synthesis (arising from reporting biases).
Certainty assessment	15	Describe any methods used to assess certainty (or confidence) in the body of evidence for an outcome.
RESULTS		
Study selection	16a	Describe the results of the search and selection process, from the number of records identified in the search to the number of studies included in the review, ideally using a flow diagram.
	16b	Cite studies that might appear to meet the inclusion criteria, but which were excluded, and explain why they were excluded.
Study characteristics	17	Cite each included study and present its characteristics.
Risk of bias in studies	18	Present assessments of risk of bias for each included study.
Results of individual studies	19	For all outcomes, present, for each study: (a) summary statistics for each group (where appropriate) and (b) an effect estimate and its precision (e.g. confidence/credible interval), ideally using structured tables or plots.
Results of syntheses	20a	For each synthesis, briefly summarise the characteristics and risk of bias among contributing studies.
	20b	Present results of all statistical syntheses conducted. If meta-analysis was done, present for each the summary estimate and its precision (e.g. confidence/credible interval) and measures of statistical heterogeneity. If comparing groups, describe the direction of the effect.
	20c	Present results of all investigations of possible causes of heterogeneity among study results.
	20d	Present results of all sensitivity analyses conducted to assess the robustness of the synthesized results.
Reporting biases	21	Present assessments of risk of bias due to missing results (arising from reporting biases) for each synthesis assessed.
Certainty of evidence	22	Present assessments of certainty (or confidence) in the body of evidence for each outcome assessed.
DISCUSSION		
Discussion	23a	Provide a general interpretation of the results in the context of other evidence.
	23b	Discuss any limitations of the evidence included in the review.
	23c	Discuss any limitations of the review processes used.
	23d	Discuss implications of the results for practice, policy, and future research.
OTHER INFORMATION		
Registration and protocol	24a	Provide registration information for the review, including register name and registration number, or state that the review was not registered.
	24b	Indicate where the review protocol can be accessed, or state that a protocol was not prepared.
	24c	Describe and explain any amendments to information provided at registration or in the protocol.
Support	25	Describe sources of financial or non-financial support for the review, and the role of the funders or sponsors in the review.
Competing interests	26	Declare any competing interests of review authors.
Availability of data, code and other materials	27	Report which of the following are publicly available and where they can be found: template data collection forms; data extracted from included studies; data used for all analyses; analytic code; any other materials used in the review.

The complete and reproducible electronic search strategies for each database are provided in Supplementary Table 2, including all keywords, Boolean operators, and controlled vocabulary terms (e.g., MeSH and Emtree). Searches were adapted to the syntax and indexing of each database and were conducted without date restrictions.

**Table 2:** Full electronic search strategies for all databases

Database	Search strategy (as executed)	Notes / Filters
PubMed	(“Clavien-Dindo Classification”[MeSH] OR “Clavien-Dindo” OR “Clavien Dindo”) AND (“postoperative complications” OR “surgical complications” OR “adverse events”) AND (“algorithm*” OR “machine learning” OR “artificial intelligence” OR “natural language processing” OR “large language model*” OR “NLP” OR “LLM” OR “GPT” OR “ChatGPT” OR “transformer”)	No date restrictions; English language
Embase	(‘clavien dindo classification’/exp OR ‘clavien dindo’) AND (‘postoperative complication’/exp OR ‘surgical complication’ OR ‘adverse event’) AND (‘algorithm’/exp OR ‘machine learning’/exp OR ‘artificial intelligence’/exp OR ‘natural language processing’/exp OR ‘large language model’ OR ‘GPT’ OR ‘ChatGPT’ OR ‘transformer’)	No date restrictions; English language
Scopus	TITLE-ABS-KEY (“Clavien-Dindo” OR “Clavien Dindo”) AND TITLE-ABS-KEY (“postoperative complication*” OR “surgical complication*” OR “adverse event*”) AND TITLE-ABS-KEY (“algorithm*” OR “machine learning” OR “artificial intelligence” OR “natural language processing” OR “large language model*” OR “NLP” OR “LLM” OR “GPT” OR “ChatGPT” OR “transformer”)	No date restrictions; English language
Cochrane Library	(“Clavien-Dindo” OR “Clavien Dindo”) AND (“postoperative complication*” OR “surgical complication*”) AND (“algorithm*” OR “machine learning” OR “artificial intelligence” OR “natural language processing” OR “large language model*” OR “NLP” OR “LLM” OR “GPT” OR “ChatGPT” OR “transformer”)	No date restrictions

Eligibility Criteria

Eligibility criteria were defined using the PICO framework, including adult patients undergoing any surgical procedure (P), automated or semi-automated algorithms for post-event detection and grading of adverse events according to the Clavien-Dindo Classification (I), manual grading or standard CDC assessment by expert reviewers as the comparator (C), and algorithm performance metrics such as agreement, accuracy, discrimination, or feasibility as outcomes (O) [6]. Only original studies reporting algorithm development, validation, or experimental evaluation were included, and studies had to specifically evaluate automated post-event CDC grading compared with expert/manual grading. Predictive-only studies, which focused solely on forecasting future complications, were excluded to ensure conceptual consistency. Additionally, studies published in languages other than English, case reports, editorials, and animal studies were excluded.

Study Selection

After removing duplicates, titles and abstracts of all identified records were screened independently by two reviewers to assess relevance based on predefined inclusion and exclusion criteria. Full texts of potentially eligible studies were retrieved and evaluated in detail for eligibility. Discrepancies between reviewers were resolved through discussion or consultation with a third reviewer. Studies were included if they reported the development, validation, or experimental evaluation of algorithms grading surgery-related adverse events according to the Clavien-Dindo Classification. Excluded studies comprised case reports, editorials, conference abstracts, and non-human studies. The final set of studies was agreed upon through consensus.

Data Extraction

Data extraction was performed independently by two reviewers using a standardized data collection form. Extracted data included study design, sample size, surgical population, type of algorithm (rule-based, machine learning, NLP/LLM), comparator (manual CDC grading), reference standard, data source (structured EHR or free-text), algorithm performance metrics (accuracy, sensitivity, specificity, Cohen’s κ, AUC), and key study outcomes. Any discrepancies were resolved through discussion or consultation with a third reviewer to ensure consistency and reliability.

Risk of Bias Assessment

The risk of bias was assessed using validated tools according to the study design. For studies developing or validating algorithms for automated grading, QUADAS-2 (adapted for algorithm validation) was used [7].To assess the risk of bias in included studies, we adapted the QUADAS-2 tool for algorithm- and LLM-based CDC grading evaluations. All four original domains (patient selection, index test, reference standard, and flow and timing) were retained, with modifications to reflect characteristics of automated grading algorithms. Specifically, signaling questions were added to capture aspects unique to algorithm validation, including handling of missing or unstructured data, use of human-in-the-loop review, model versioning and prompt documentation, external validation across different sites, and data de-identification procedures. Risk-of-bias judgments were defined as low, unclear, or high based on pre-specified thresholds for each domain, for example, low risk if the index test was externally validated with blinded manual grading, high risk if there was incomplete reporting of algorithm performance or selective inclusion of cases. For studies employing machine learning prediction models, PROBAST was applied [8]. Domains assessed included patient selection, index test (algorithm), reference standard, flow and timing, predictors, outcomes, and analysis. Each domain was rated as low, moderate, or high risk of bias, with justification recorded. Disagreements were resolved by consensus among the reviewers.

Data Synthesis

Given the heterogeneity in algorithm types, surgical populations, and outcome measures, a narrative synthesis was performed. Descriptive statistics were used to summarize study characteristics and algorithm performance metrics. Agreement between automated and manual CDC grading was reported as Cohen’s κ or ICC, where available. Predictive performance of machine learning models was summarized using AUC, sensitivity, specificity, and overall accuracy. Comparisons across studies focused on algorithm type, data source, validation method, and generalizability to different surgical settings. Studies were grouped thematically by algorithm type (rule-based vs LLM-based), data source, and performance metrics. A pilot extraction was conducted on a subset of studies to refine the extraction form.

Registration

This systematic review was conducted in accordance with the PRISMA 2020 guidelines. The review protocol was not registered in PROSPERO or any other publicly accessible registry.

Results

Study Selection Process

**Figure 1:**
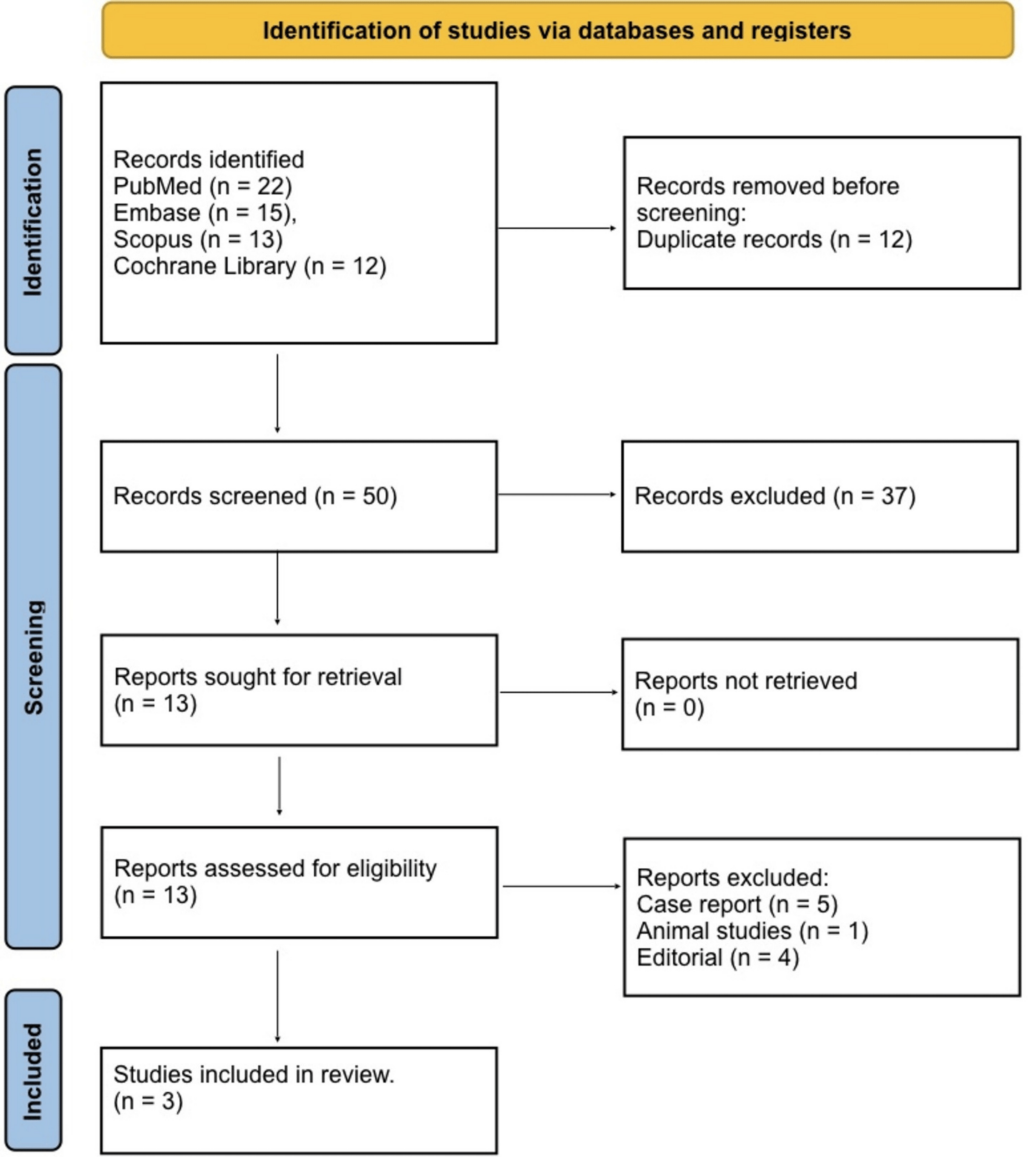
PRISMA 2020 flow diagram

Figure 1 shows that a total of 62 records were identified through database searching, including 22 from PubMed, 15 from Embase, 13 from Scopus, and 12 from the Cochrane Library. After removing 12 duplicate records, 50 unique studies remained for title and abstract screening. Following this initial screening, 37 studies were excluded as they did not meet the inclusion criteria based on relevance or study design. The full texts of 13 articles were retrieved and assessed for eligibility. Of these, 10 reports were excluded, comprising five case reports, one animal study and four editorials, leaving a total of three studies that met all inclusion criteria and were included in the final qualitative synthesis.

Characteristics of the Selected Studies

In Table 3, three studies evaluated algorithms for grading surgery-related adverse events (AEs) according to the Clavien-Dindo Classification (CDC). Olsson et al. developed and validated a fully automated, rule-based algorithm using structured electronic health record (EHR) data from 1,379 elective colorectal procedures, with a random sample of 399 manually reviewed by a colorectal surgeon and research nurse; the algorithm demonstrated substantial to almost perfect agreement with human graders, achieving Cohen’s κ of 0.77 versus surgeon and up to 0.89 after correcting annotation errors, with an intraclass correlation coefficient (ICC) for the Comprehensive Complication Index (CCI) of 0.89 [9]. Alcı et al. applied supervised machine learning (ML) models, evaluating 11 classifiers on 179 patients undergoing epithelial ovarian cancer surgery, and found that a Bayesian network best predicted severe (CDC ≥ III) complications with an area under the curve (AUC) of 0.863, sensitivity 76.9%, and overall accuracy 82.2%, demonstrating potential for pre- and intraoperative risk stratification [10]. Staubli et al. tested a large language model (LLM)-based natural language processing (NLP) tool on both generated clinical vignettes and real discharge summaries across multiple surgical specialties; the LLM extracted and graded complications from unstructured free-text with very high agreement compared to expert clinician panels, achieving ~97% accuracy on examples and Cohen’s κ of 0.92 on real discharge summaries, outperforming earlier LLM versions [11]. Together, these studies highlight CDC grading using rule-based algorithms, ML, and LLM/NLP approaches, with applications ranging from real-time EHR surveillance to predictive risk modeling and research data extraction. The included studies covered heterogeneous surgical domains, limiting direct comparability.

**Table 3:** Characteristics of the selected studies AE = Adverse event; CDC = Clavien-Dindo Classification; CCI = Comprehensive Complication Index; EHR = Electronic health record; ML = Machine learning; LLM = Large language model; NLP = Natural language processing; AUC = Area under the curve.

Author (year)	Study design	(population / outcome)	Intervention (algorithm)	Comparator	Main finding (short)	Surgical setting	Algorithm type	Data source	Reference standard	Performance (reported)	Important / outcome
Olsson LB et al., 2025 [9]	Retrospective development + validation on registry; random sample validation (n=1379 procedures; sample n=399 reviewed)	Elective colorectal procedures; AEs within 30 days graded by Clavien–Dindo	Fully automated algorithm that returns a C-D grade per AE (rules applied to EHR events)	Manual annotation by colorectal surgeon (gold standard) and research nurse (current practice)	Algorithm showed substantial-to-almost-perfect agreement with human graders; improved after correcting annotation errors	Elective colorectal surgery (single tertiary centre)	Rule-based / EHR-driven automated grading algorithm	Full hospital EHR (structured events, procedures, medications, notes)	Colorectal surgeon (gold standard); research nurse	Cohen’s κ = 0.77 (alg vs surgeon); κ = 0.74 (alg vs nurse); κ up to 0.89 after correction; ICC for CCI = 0.89 after correction.	Demonstrates feasibility of automated CDC grading from EHR; supports real-time surveillance and possible implementation with continuous evaluation.
Alcı A et al., 2025 [10]	Single-centre retrospective ML development + internal test split (n=179; train 134 / test 45)	Patients undergoing epithelial ovarian cancer surgery; aim = predict Clavien–Dindo ≥ III	Machine-learning models evaluated (11 classifiers); final best = Bayesian network (BayesNet) predicting CDC ≥ III	Actual CDC grades from charted postoperative outcomes (chart review)	BayesNet achieved highest discrimination among tested models (good predictive ability for Grade ≥ III)	Epithelial ovarian cancer cytoreductive surgery (gynaecologic oncology)	Supervised ML (Bayesian network best of 11 models)	Clinical + intraoperative variables (49 predictors: demographics, comorbidities, intraop findings, labs)	Chart review / actual CDC grade from patient records	AUC = 0.863 (p<0.001); TP rate 76%; FP rate 15.6%; sensitivity ≈ 76.9%; overall accuracy 82.2%.	Shows ML can predict serious (≥III) CDC complications pre-/intra-operatively; may assist risk stratification and perioperative planning in oncology surgery.
Staubli S et al., 2024 [11]	Experimental validation study of LLM/NLP tool on generated scenarios and real discharge summaries; comparison with experts	Multiple clinical vignettes and real discharge summaries from post-op patients; test of CDC grading capability	Large language model used to extract & grade complications per CDC from free-text	Human expert graders (panels of clinicians)	4 mirrored CDC definitions well; very high agreement with human grading on real discharge summaries	Mixed surgical specialties / real-world discharge summaries (multicentre / multi-specialty data in examples)	NLP / large language model (LLM)	Unstructured free-text (generated clinical examples + real discharge summaries)	Human experts (clinician panel)	For single complications: ~97% accuracy (examples); real discharge summaries: Cohen’s κ = 0.92 (95% CI 0.82–1.00). ChatGPT-4 outperformed 3.5.	Demonstrates high potential of LLM-based NLP to extract and grade CDC from free text with near-expert agreement; suggests value for automating chart review and research extraction but need governance

Risk of Bias Assessment

Table 4 shows, the risk of bias for the included studies was assessed using QUADAS-2 for algorithm and LLM/NLP validation studies and PROBAST for the ML prediction study. Olsson et al. demonstrated low risk of bias across all domains, including patient selection, index test, reference standard, and flow and timing, as consecutive elective colorectal surgery cases were clearly defined, the automated CDC algorithm was applied uniformly and blinded to reference grading, and all patients were assessed without missing data [9]. In contrast, Alcı et al. showed low risk in participants and outcome domains, but high risk in predictors and analysis due to the inclusion of 49 variables with unclear blinding and a small sample size (n=179) with internal validation only, which may increase overfitting [10]. Staubli et al. had low risk of bias in index test, reference standard, and flow and timing, but moderate risk in patient selection because synthetic vignettes were included, which may reduce representativeness, although LLM-based CDC grading was applied consistently and compared against an independent expert clinician panel [11]. Overall, one study demonstrated low risk of bias across all domains, while the remaining study showed moderate concerns primarily related to data representativeness.

**Table 4:** Risk of bias assessment CDC = Clavien-Dindo Classification; LLM = Large language model; NLP = Natural language processing; ML = Machine learning.

Study (Year)	Assessment Tool	Domain	Risk of Bias	Justification
Olsson LB et al., 2025 [9]	QUADAS-2 (adapted for algorithm validation)	Patient Selection	Low	Consecutive elective colorectal surgery cases; clearly defined inclusion/exclusion criteria; no selective sampling.
		Index Test (Automated CDC Algorithm)	Low	Algorithm rules predefined and applied uniformly; index test blinded to reference grading.
		Reference Standard	Low	Surgeon-reviewed manual grading used as gold standard; independent and blinded to algorithm output.
		Flow & Timing	Low	All patients assessed with both algorithm and human grading; no missing data; identical assessment window.
Alcı A et al., 2025 [10]	PROBAST (ML prediction model)	Participants	Low	Clearly defined ovarian cancer cohort; appropriate for predictive modelling.
		Predictors	High	49 predictors included; unclear blinding; potential information leakage increases bias.
		Outcome (CDC ≥ III)	Low	Outcomes objectively defined using Clavien–Dindo; extracted consistently from chart review.
		Analysis	High	Small sample size (n=179) risks overfitting; internal validation only; no external dataset.
Staubli S et al., 2024 [11]	QUADAS-2 (adapted for LLM/NLP validation)	Text/Patient Selection	Moderate	Includes synthetic vignettes and real summaries; synthetic data reduces representativeness.
		Index Test (LLM-based CDC Grading)	Low	LLM applied consistently; predefined extraction and grading workflow with no manual intervention.
		Reference Standard	Low	Independent clinician expert panel; strong unbiased reference.
		Flow & Timing	Low	All summaries processed by LLM and experts; complete data; consistent timing across assessments.

Discussion

This review is the first to systematically evaluate automated CDC grading tools specifically focused on post-event classification. Review addresses a critical unmet need in surgical quality monitoring: the automation of Clavien-Dindo Classification (CDC) grading. Traditionally, the CDC has been applied through manual chart review, which is resource‑intensive, inconsistent, and subject to inter‑observer variability. Indeed, despite its widespread adoption, the original Clavien-Dindo classification has been shown to have nontrivial variability even among experienced clinicians; early validation work demonstrated that agreement across centers for difficult case scenarios was only around 89% and that perceptions of complication severity could differ among patients, nurses, and physicians [12]. Moreover, in certain specialties such as head and neck surgery, moderate interobserver reliability has been reported, underlining the subjective challenges inherent in manual grading [13]. By synthesizing studies that apply automated algorithms, including rule‑based systems, machine learning (ML), and large language model (LLM)/NLP approaches, this review demonstrates how rule-based and LLM-based approaches may help standardize CDC grading and make real-time surveillance feasible.

For instance, the rule-based algorithm applied in the EHR of colorectal surgery achieved substantial agreement with human graders (Cohen’s κ up to 0.89 after correction and ICC of 0.89 for the Comprehensive Complication Index) [9]. This kind of performance supports the feasibility of deploying automated grading in practice, potentially reducing the burden on clinical reviewers and improving consistency in complication reporting. Machine‑learning models, like the Bayesian network trained on 49 clinical and intraoperative predictors in ovarian cancer surgery, further extend the value by enabling the prediction of high-grade (CDC ≥ III) complications before they occur. In that study, the model achieved an AUC of 0.863 with good sensitivity and an overall accuracy of ~82%. Such predictive capacity could be transformative: it enables risk stratification, informs perioperative planning, and may guide resource allocation (e.g., higher‑risk patients could receive more intensive monitoring or prophylactic interventions).

This aligns with broader trends in surgical risk modeling; for example, interpretable ML models have recently been developed in rectal cancer to predict CDC grade III/IV complications, identifying hematological markers as significant predictors [14]. Similarly, in laryngeal cancer surgery, a random forest model achieved very high discriminative performance (AUC ~0.84 in test set), with SHAP-based feature analysis revealing clinically intuitive variables (nutritional status, tumor subsite, vocal cord mobility) as key contributors [15]. On the other hand, LLM/NLP‑based tools tackle a different and equally important problem: the unstructured nature of much of the clinical documentation. Free-text discharge summaries and clinical notes are rich in nuance, but extracting structured information for grading is cumbersome. The study using ChatGPT (or more broadly, an LLM) demonstrated ~97% accuracy on synthetic vignettes and a Cohen’s κ of 0.92 when applied to real discharge summaries [11]. This near-expert level of concordance suggests that LLMs can reliably interpret narrative clinical data, potentially unlocking large-scale retrospective research, multicenter audits, and real-time complication surveillance without needing manual abstraction.

Importantly, by comparing across these algorithmic approaches, our review highlights key trade‑offs: rule-based systems may be more transparent and easier to validate but may struggle with rare or complex events; ML models provide predictive power but require good-quality structured data and carry a risk of overfitting; LLM/NLP tools can interpret unstructured text but demand robust governance (e.g., privacy protection, interpretability). The diversity of approaches signals that no single method is universally best, and hybrid strategies might be optimal depending on the setting and available data. From a clinical and systems‑level perspective, the adoption of automated CDC grading has major implications. First, for quality improvement programs, automated tools can enable continuous, scalable monitoring of complications, reducing reliance on periodic manual audits. Second, for research, these tools can facilitate large-scale data extraction from EHRs and clinical notes, improving the efficiency and accuracy of surgical outcomes research. Third, from a patient safety standpoint, early detection and grading of complications in real time could trigger alerts, prompting interventions that reduce morbidity and readmission rates.

In addition, there is growing precedent for integrating predictive risk models into clinical workflows. For example, an ML model for predicting severe complications after cytoreductive surgery with hyperthermic intraperitoneal chemotherapy (CRS-HIPEC) was externally validated with modest discriminative performance (AUC ~0.65 in external cohorts) [16]. Such real-world validation underscores the feasibility and clinical utility of AI-driven risk stratification tools, lending further support to the potential of automated CDC grading beyond retrospective research. However, the path to clinical implementation is not without challenges. Data heterogeneity across centers, varying EHR systems, local documentation practices, and differences in patient populations may affect algorithm performance and generalizability. There is also a need for rigorous prospective evaluation: how will automated grading affect decision-making, resource utilization, and clinical outcomes? Additionally, ethical and regulatory concerns, including patient privacy, data security, algorithmic transparency, and accountability, must be addressed.

This review is limited by the small number of included studies (n = 3), substantial heterogeneity in surgical populations, algorithm types, and outcome measures, and the predominance of single-center retrospective designs with limited external validation, which restricts generalizability and precludes meta-analysis. In addition, variability in reference standards and the inherent subjectivity of manual Clavien-Dindo grading may have influenced reported performance, and LLM-based studies partly relied on synthetic data, reducing real-world representativeness. Additionally, the Clavien-Dindo system itself has inherent limitations, including ambiguity in grading borderline cases, reliance on therapeutic decisions rather than physiological severity, and variability in interpretation across surgical specialties. The search was not updated immediately prior to submission. Consequently, very recent studies on AI/LLM-based CDC grading may not have been captured. However, the chosen search end date reflects the time point at which data extraction was feasible. Future research should focus on multicentre prospective studies with standardized reference standards, robust external validation, and transparent reporting of algorithm development. Integration of hybrid approaches combining structured EHR data with NLP/LLM methods, along with evaluation of real-world clinical impact on quality improvement, workflow efficiency, and patient outcomes, will be essential before widespread clinical implementation.

## Conclusions

Automated algorithms for grading surgery-related adverse events according to the Clavien-Dindo Classification demonstrate promising but preliminary reliability, accuracy, and near-expert concordance. Rule-based, machine learning, and LLM/NLP approaches enable consistent, scalable, and efficient complication grading from both structured and unstructured data sources. These tools have significant potential to transform postoperative care by facilitating real-time surveillance, predictive risk stratification, research data extraction, and continuous quality improvement. With further multicenter validation, integration into clinical workflows, and evaluation of patient-centered outcomes, automated CDC grading can enhance surgical safety, efficiency, and overall healthcare quality across specialties.
